# Association of parity with birthweight and neonatal death in five sites: The Global Network’s Maternal Newborn Health Registry study

**DOI:** 10.1186/s12978-020-01025-3

**Published:** 2020-12-17

**Authors:** Ana Garces, Wilton Perez, Margo S. Harrison, Kay S. Hwang, Tracy L. Nolen, Robert L. Goldenberg, Archana B. Patel, Patricia L. Hibberd, Adrien Lokangaka, Antoinette Tshefu, Sarah Saleem, Shivaprasad S. Goudar, Richard J. Derman, Jacquelyn Patterson, Marion Koso-Thomas, Elizabeth M. McClure, Nancy F. Krebs, K. Michael Hambidge

**Affiliations:** 1 Instituto de Nutrición de Centroamérica y Panamá, Guatemala City, Guatemala; 2 University of Colorado School of Medicine, Denver, CO USA; 3 RTI International, Durham, NC USA; 4 Department of Obstetrics and Gynecology, Columbia University School of Medicine, New York, NY USA; 5 Lata Medical Research Foundation, Nagpur, India; 6 Boston University School of Public Health, Boston, MA USA; 7 Kinshasa School of Public Health, DRC, Kinshasa, Congo; 8 Aga Khan University, Karachi, Pakistan; 9 KLE Academy Higher Education and Research J N Medical College Belagavi, Karnataka, India; 10 Thomas Jefferson University, Philadelphia, USA; 11 University of North Carolina at Chapel Hill, Chapel Hill, USA; 12 Eunice Kennedy Shriver National Institute of Child Health and Human Development, Bethesda, MD USA

**Keywords:** Nulliparity, Birth weight, Neonatal death, Pregnancy outcomes, Global network

## Abstract

**Background:**

Nulliparity has been associated with lower birth weight (BW) and other adverse pregnancy outcomes, with most of the data coming from high-income countries. In this study, we examined birth weight for gestational age z-scores and neonatal (28-day) mortality in a large prospective cohort of women dated by first trimester ultrasound from multiple sites in low and middle-income countries.

**Methods:**

Pregnant women were recruited during the first trimester of pregnancy and followed through 6 weeks postpartum from Maternal Newborn Health Registry (MNHR) sites in the Democratic Republic of Congo (DRC), Guatemala, Belagavi and Nagpur, India, and Pakistan from 2017 and 2018. Data related to the pregnancy and its outcomes were collected prospectively. First trimester ultrasound was used for determination of gestational age; (BW) was obtained in grams within 48 h of delivery and later transformed to weight for age z-scores (WAZ) adjusted for gestational age using the INTERGROWTH-21st standards.

**Results:**

15,121 women were eligible and included. Infants of nulliparous women had lower mean BWs (males: 2676 gr, females: 2587 gr, total: 2634 gr) and gestational age adjusted weight for age z-scores (males: − 0.73, females: − 0.77, total: − 0.75,) than women with one or more previous pregnancies. The largest differences were between zero and one previous pregnancies among female infants. The associations of parity with BW and z-scores remained even after adjustment for maternal age, maternal height, maternal education, antenatal care visits, hypertensive disorders, and socioeconomic status. Nulliparous women also had a significantly higher < 28-day neonatal mortality rate (27.7 per 1,000 live births) than parous women (17.2 and 20.7 for parity of 1–3 and ≥ 4 respectively). Risk of preterm birth was higher among women with ≥ 4 previous pregnancies (15.5%) compared to 11.3% for the nulliparous group and 11.8% for women with one to three previous pregnancies (p = 0.0072).

**Conclusions:**

In this large sample from diverse settings, nulliparity was independently associated with both lower BW and WAZ scores as well as higher neonatal mortality compared to multiparity.

## Background

The Maternal Newborn Health Registry (MNHR) is a prospective, population-based registry of pregnancies and deliveries conducted under the auspices of the Global Network for Women’s and Children’s Health Research (GN), a multi-country research network funded by the *Eunice Kennedy Shriver* National Institute of Child Health and Human Development (NICHD) [1, 2]. The primary purpose of the MNHR is to quantify and analyze trends in pregnancy outcomes over time [3, 4].

Neonates born to nulliparous women are reported to be at higher risk of death and other adverse outcomes, including low BW, being small for gestational age, and prematurity [5, 6]. A meta-analysis that reviewed data from Asia, Africa and Latin America found that these risks were highest among nulliparous women under 18 years of age in comparison to women with one or two previous pregnancies between 18 and 35 years of age [7]. In addition to maternal age, associations for risk of adverse pregnancy outcomes have been found with parity for maternal height, maternal education, number of antenatal care visits and for newborn sex [8, 9].

Neonates born to multiparous women have been found to have higher BWs and better weight gain during infancy [10]. However, these positive effects have been limited to the second or third pregnancies and are associated with younger maternal age. Women with parity over three and age 35 or older have been found to have higher odds of adverse outcomes in low and middle-income settings [7].

The higher risks that infants of nulliparous women face may be associated with factors related to immaturity of the mother: incomplete growth, small size of the uterus and fetal competition for nutrients [7, 11]. Although the exact mechanisms are not clear, it seems that the first pregnancy “primes” the body for subsequent ones.

In this paper, our aim is to determine the association between parity and BW among women in five research sites of the MNHR. Because of its cohort design, large sample size, multiple sites and the quality of the data (including gestational age dating with first trimester ultrasound, implemented broadly in 2017), the MNHR has the potential to extend the current knowledge of the importance of parity on fetal growth and other offspring outcomes.

## Methods

### Study design and population

Participants were recruited from MNHR sites in the Democratic Republic of Congo (DRC), Guatemala, Belagavi and Nagpur, India, and Pakistan, between January 2017 and December 2018. All pregnant women living in the defined geographic areas were enrolled in the MNHR during pregnancy and followed from consent until 6 weeks after delivery. Data related to the pregnancy and its outcomes were collected prospectively.

For this analysis, we included women with a first trimester ultrasound examination for determination of gestational age. Exclusions included cases with a maternal death prior to labor/delivery, miscarriage, medical termination of pregnancy, stillbirth, multiple pregnancy, and missing birthweight for gestational age z-score due to missing infant sex, birthweights missing or captured more than 3 days post birth, or gestational age less than 24 or more than 42 weeks. Parity was categorized as: zero (nulliparous), one to three, and four or more previous pregnancies. Maternal height was measured using a stadiometer or scale. Because a large amount of first trimester weight data was missing, neither maternal weight nor body mass index were included in these analyses. Hypertensive disorders were defined as any evidence of hypertensive disease or severe preeclampsia or eclampsia, including elevated blood pressure, proteinuria and/or seizures. The socioeconomic status index of the household was calculated from 9-items that combine housing conditions and household assets [12]. The index outcomes ranged from 0 (poorest) to 100 (least poor). Gestational age was determined using data from a first-trimester ultrasound. BW was obtained in grams and later transformed to z-scores adjusted for gestational age using the INTERGROWTH-21st standards [https://intergrowth21.tghn.org/]. Neonatal mortality was reported up to 28 days of life.

### Data analysis

Numerical and categorical data were summarized using mean with standard deviation and proportion respectively. Adjusted mean estimates with 95% confidence interval of both BW and BW z-scores across parity levels were obtained using linear mixed models controlling for the sampling cluster as a random effect. The analysis was adjusted by site, maternal age, height, maternal education, antenatal care, hypertensive disorders, and socioeconomic status. Models were presented by the child’s sex and total sample. Separate interaction terms between parity and other variables (maternal age, height, care visits, education, and socioeconomic status) were explored. The associations of parity with mortality and preterm births were assessed via Cochran–Mantel–Haenszel tests stratified by cluster. P-value < 0.05 was considered as significant.

### Ethical issues

The institutional review boards and ethics committees at the participating study sites (Kinshasa School of Public Health, Kinshasa, DRC; INCAP, Guatemala City, Guatemala; KLE University's JN Medical College, Belagavi, India; Lata Medical Research Foundation, Nagpur, India; and Aga Khan University, Karachi, Pakistan), their affiliated US partner institutions (University of North Carolina at Chapel Hill, University of Colorado, Thomas Jefferson University, Boston University, Columbia University, University of Indiana, and the data coordinating center (RTI International) approved the study. Each woman enrolled provided informed consent regarding the use of data related to her pregnancy, including minors 14–17 years of age in countries where married or pregnant minors (or their authorized representatives) are legally permitted to give consent. All research staff in each country responsible for obtaining consent were trained and certified in matters related to human protection and ethical procedures. Names of women and other personally identifiable information were excluded in the analytical database.

## Results

**Fig. 1:**
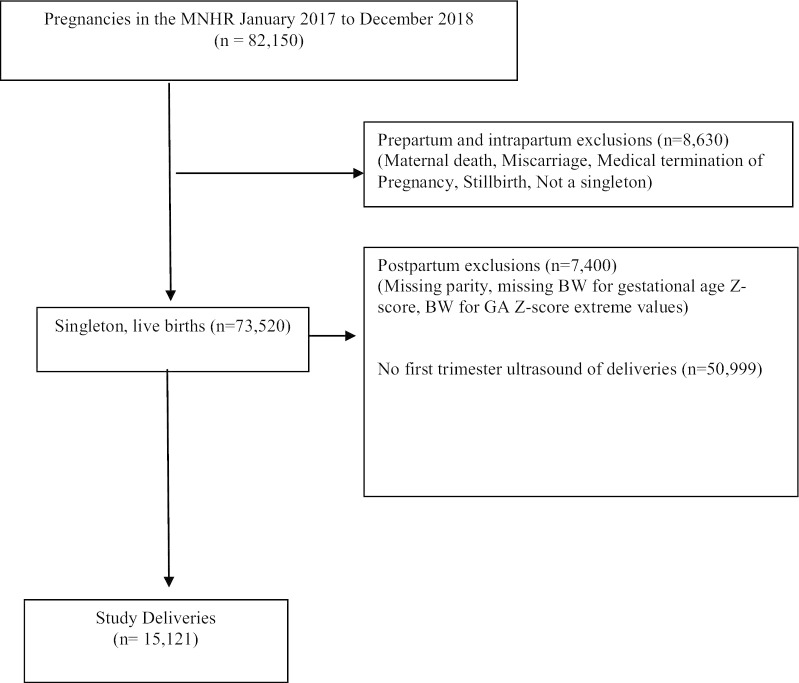
Enrollment flow diagram

Of the 82,150 deliveries that occurred in participating sites of the MNHR between January 2017 and December 2018, 8630 were excluded due to multiple pregnancy, medical termination of pregnancy, stillbirth. Most exclusions (n = 50,999) were due to lack of first trimester ultrasound, resulting in 15,121 (18.4%) deliveries that met the inclusion criteria for analysis (Fig. 1). Overall, nulliparous women made up 57.8% of the sample, women with one to three previous pregnancies accounted for 37.7%, and women with four or more previous pregnancies, 4.5%. Site differences were observed in the distribution of parity with 39.7% of nulliparous women enrolling at the Belagavi site and the DRC and Pakistan sites contributing the most women with parity ≥ 4 (39.8% and 36.4% respectively). In the study sample, nulliparous women were younger and shorter, had received more education than parous women combined and had a higher prevalence of hypertensive diseases/severe preeclampsia/eclampsia (p-values < 0.01) (Table 1). Due to observed differences in sites regarding the distribution of parity, site was adjusted for in all analyses assessing the association of parity with BW. However, site was no longer significant after adjusting for these maternal characteristics.

**Table 1:** Maternal characteristics and outcomes by parity

	Nulliparous	Parous		Total	Nulliparous vs. Parous p-value^a^
		1 to 3	4 or more		
Total sample size	8742	5703	676	15,121	
Study site, N (%)					< 0.0001
DRC	974 (11.1)	503 (8.8)	269 (39.8)	1746 (11.5)	
Guatemala	1344 (15.4)	673 (11.8)	83 (12.3)	2100 (13.9)	
Belagavi, India	3473 (39.7)	3,546 (62.2)	77 (11.4)	7096 (46.9)	
Nagpur, India	1748 (20.0)	455 (8.0)	1 (0.1)	2204 (14.6)	
Pakistan	1203 (13.8)	526 (9.2)	246 (36.4)	1975 (13.1)	
Maternal age, N (%)					< 0.0001
< 20	2965 (33.9)	145 (2.5)	1 (0.1)	3111 (20.6)	
20–35	5753 (65.8)	5466 (95.8)	560 (82.8)	11,779 (77.9)	
≥ 36	24 (0.3)	92 (1.6)	115 (17.0)	231 (1.5)	
Maternal height, N (%) (cm)					0.0024
< 145	835 (9.6)	453 (7.9)	37 (5.5)	1325 (8.8)	
145–155	5292 (60.5)	3587 (62.9)	322 (47.6)	9201 (60.9)	
≥ 156	2614 (29.9)	1663 (29.2)	317 (46.9)	4594 (30.4)	
Maternal education, N (%)					< 0.0001
No formal education	1308 (15.0)	840 (14.7)	391 (57.8)	2539 (16.8)	
Primary/secondary	6063 (69.4)	4248 (74.5)	279 (41.3)	10,590 (70.0)	
University	1370 (15.7)	615 (10.8)	6 (0.9)	1991 (13.2)	
Antenatal care visits, Mean (SD)	4.9 (1.8)	4.7 (1.7)	4.2 (2.0)	4.8 (1.8)	< 0.001
Socioeconomic status score, N (%)					
< 34	1135 (13.9)	582 (11.3)	314 (50.3)	2031 (14.6)	0.8085
34–65	3360 (41.1)	2202 (42.8)	235 (37.7)	5797 (41.6)	
≥ 66	3677 (45.0)	2360 (45.9)	75 (12.0)	6112 (43.8)	
Hypertensive disease/severe preeclampsia/eclampsia, N (%)	498 (5.7)	150 (2.6)	19 (2.8)	667 (4.4)	< 0.0001

^a^p-values for categorical maternal characteristics are based on Cochran–Mantel–Haenszel tests of each characteristic and nulliparous vs. parous stratified by cluster, while p-values for continuous maternal characteristics are based on t-tests comparing the difference in the means of nulliparous vs. parous for each characteristic

Nulliparous women had 1.5 times higher rates of low BW (< 2500 g) infants than parous women (Table 2). Infants of nulliparous women had a significantly higher 28-day mortality rate (27.7 per 1000 live births) than women with one to three (17.2 per 1000) and four or more (20.7 per 1000) pregnancies (p = 0.0006). There was also a significantly higher proportion of preterm infants in women with four and more previous pregnancies. 15.5% of infants born to these women were preterm compared to 11.3% for the nulliparous group and 11.8% for women with one to three previous pregnancies (p = 0.0072).

**Table 2:** Neonatal characteristics by parity

	Nulliparous	Parous		Total	Nulliparous vs. parous p-value^a^
		1 to 3	4 or more		
Study population	8742	5703	676	15,121	
Gender, N (%)					0.0703
Male	4551 (52.1)	2914 (51.1)	335 (49.6)	7800 (51.6)	
Female	4191 (47.9)	2789 (48.9)	341 (50.4)	7321 (48.4)	
Gestational age, N (%)					0.0072
Preterm (< 37 weeks)	992 (11.3)	674 (11.8)	105 (15.5)	1771 (11.7)	
Term (≥ 37 weeks)	7750 (88.7)	5029 (88.2)	571 (84.5)	13,350 (88.3)	
Birth weight categories, N (%) (g)					< 0.0001
< 1500	72 (0.8)	40 (0.7)	4 (0.6)	116 (0.8)	
1500–2000	470 (5.4)	175 (3.1)	28 (4.1)	673 (4.5)	
2001–2500	2054 (23.5)	922 (16.2)	101 (14.9)	3077 (20.3)	
≥ 2501	6146 (70.3)	4566 (80.1)	543 (80.3)	11,255 (74.4)	
Mean (SD)	2738.7 (445.8)	2840.4 (427.5)	2907.4 (478.6)	2784.6 (444.0)	< 0.0001
Neonatal deaths < 28 days, N (Rate /1000)	242 (27.7)	98 (17.2)	14 (20.7)	354 (23.4)	0.0006

^a^p-values for categorical maternal characteristics are based on Cochran–Mantel–Haenszel tests of each characteristic and nulliparous vs. parous stratified by cluster, while p-values for continuous maternal characteristics are based on t-tests comparing the difference in the means of nulliparous vs. parous for each characteristic

In a model controlling for cluster as a random effect and adjusting for site, parity, maternal age, height, education, hypertensive disorders, antenatal care and socio-economic status, nulliparous also had significantly lower mean BWs (Table 3). Increases in BW were associated with increasing parity up to four or more previous pregnancies overall and for male infants; the largest increases overall and for males (64 g and 76 g, respectively) occurred between the infants of nulliparous women and women with one previous pregnancy. Female infant birthweight was over 50 g greater between infants of nulliparous women and women with one previous pregnancy as well as between infants of women with three versus two previous pregnancies.

**Table 3:** Adjusted estimated BW (95% CI), in grams, by parity

	Male		Female		Total	
	N	Mean BW	N	Mean BW	N	Mean BW
Nulliparous	4245	2676 (2636, 2717)	3914	2587 (2546, 2628)	8159	2634 (2604, 2663)
1	1669	2752 (2706, 2798)	1567	2643 (2596, 2690)	3236	2698 (2664, 2732)
2	684	2777 (2725, 2830)	656	2675 (2622, 2728)	1340	2725 (2687, 2764)
3	270	2803 (2738, 2867)	288	2737 (2674, 2800)	558	2766 (2721, 2812)
4 or more	302	2823 (2763, 2883)	313	2727 (2669, 2786)	615	2774 (2731, 2817)
p-value		< 0.0001		< 0.0001		< 0.0001

Estimated birthweight and p-values overall and by infant sex are from a linear mixed model with BW as the outcome controlling for cluster as a random effect and adjusting for site, parity, maternal age, height, education, hypertensive disorders, antenatal care and socio-economic status

**Fig. 2:**
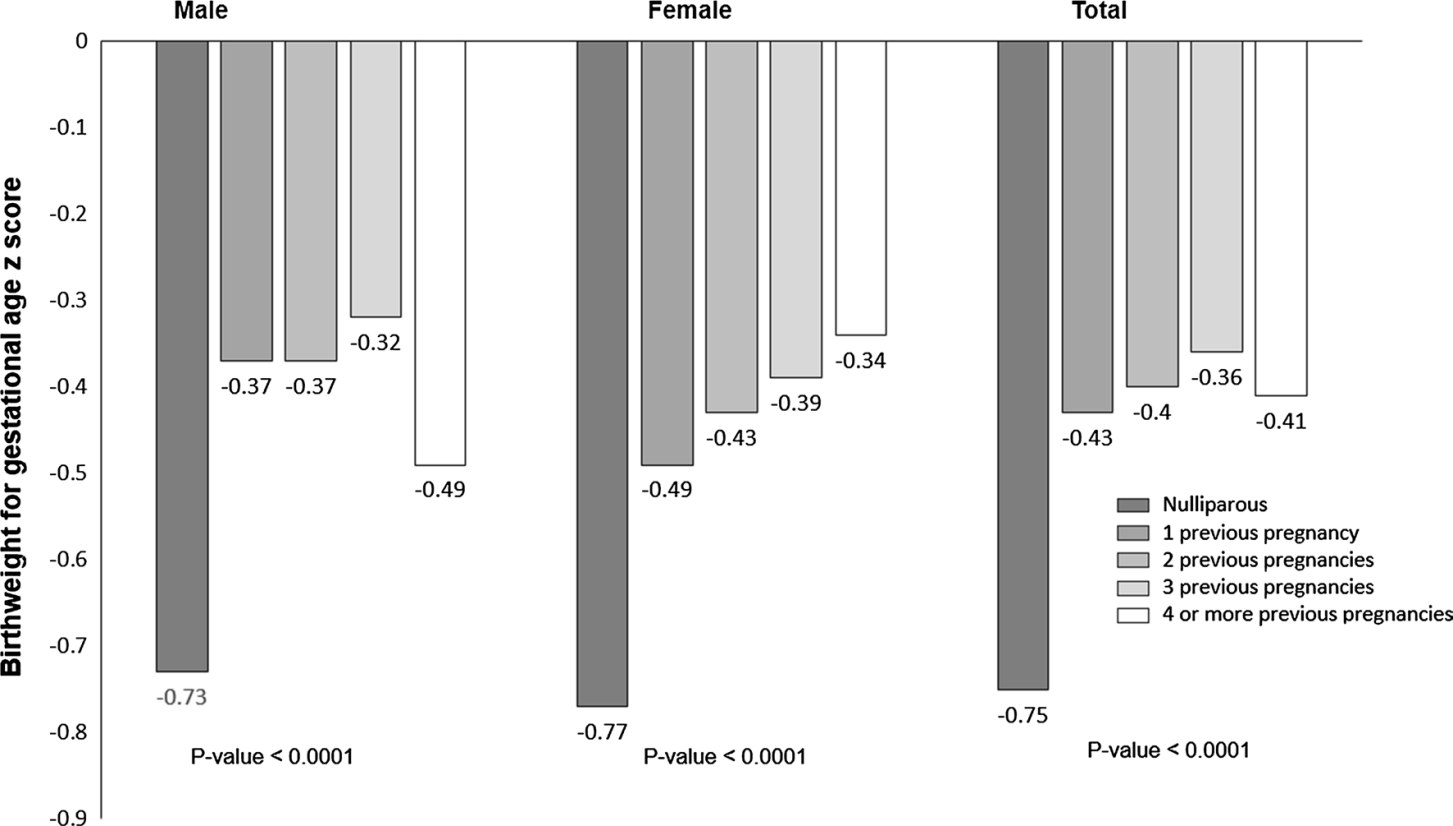
Adjusted birth weight for gestational age z-scores and parity by infant sex. Estimated BW for gestational age z-scores and p-values overall and by infant sex are from a linear mixed model with z-score as the outcome controlling for cluster as a random effect and adjusting for site, parity, maternal age, height, education, hypertensive disorders, antenatal care, and socio-economic status

The adjusted BW for gestational age z-scores (WAZ) by parity, categorized by infant sex, are presented in Fig. 2. The WAZ score of infants of nulliparous women were significantly lower than those of women with one, two or three previous pregnancies in male infants, in female infants, and overall (based on non-overlapping confidence intervals, results not shown). The largest incremental differences in z-scores were observed between infants of nulliparous women and women with one previous pregnancy overall as well as for male and female infants (0.36, 0.28, and 0.32, respectively). Overall, increases in WAZ scores were associated with increasing parity up to three previous pregnancies. There was no interaction of parity with maternal age, height, education antenatal care visits, or socioeconomic status on the association with BW for gestational age z-scores.

## Discussion

In line with previous studies, the nulliparous women in this study of five low and middle-income country sites had lower mean BWs and WAZ scores than women with one, two or three previous pregnancies [10, 13, 14]. We also found the largest incremental differences between zero and one pregnancies. However, we did not find interactions between parity and the characteristics of maternal age, maternal height, antenatal care visits, socioeconomic status, or maternal education with respect to the association with parity (results not shown).

The higher neonatal mortality rates in our study among infants of nulliparous women and the higher proportion of preterm infants in women with four or more pregnancies are also similar to results from previous reports [7]. This is important information for choosing antenatal care in the perinatal period, as it indicates the vulnerability that nulliparous women face, independent of other risk factors. This large, multi-site cohort study strengthens the existing knowledge by including the analysis of other covariates in nulliparous women of low and middle-income settings.

Because of the multiple sites and large number of participants, the MNHR has the potential to extend the current knowledge of the importance of parity on fetal growth and other pregnancy outcomes [5, 7]. This study provides quantitative data on differences in BW, preterm birth, and neonatal mortality by parity for five low-income sites. Additionally, the effect of nulliparity independent from age, which differs from prior studies, adds to the evidence base. These results can inform public health strategies geared both at nulliparous women and those with greater than four prior pregnancies residing in high-risk settings.

The strengths of this study include the ultrasound determination of gestational age that allowed for an accurate estimation of BW for gestational age—adjusted z scores and preterm birth. However, the limited availability of first trimester ultrasound reduced our sample size and resulted in inclusion of less than one-fourth of the women participating in the MNHR for this analysis. This may have biased our results in favor or women who enrolled in the MNHR in the first trimester. Strengths of the study included the large sample size, multiple sites on 3 continents, and standard data collection methodologies.

## Conclusions

This analysis confirms that in low and middle-income countries, the infants of nulliparous women have lower BWs and higher neonatal mortality than women with one to four previous pregnancies. The association between parity and birthweight was not affected by maternal age, height, education, antenatal care visits, hypertensive disorders, or socioeconomic status. These results contribute to the evidence base on pregnancy outcomes and may have implications for the targeting of nutritional and reproductive interventions aimed at improving pregnancy outcomes in low and middle-income settings.

## Data Availability

Data from the study will be available at the NICHD data repository (N-DASH): https://dash.nichd.nih.gov/.

## Abbreviations

BW: Birth weight
MNHR: Maternal Newborn Health Registry
DRC: Democratic Republic of Congo
WAZ: Weight for age z-scores
GN: Global Network for Women’s and Children’s Health Research
NICHD: *Eunice Kennedy Shriver* National Institute of Child Health and Human Development
